# Influence of the atrio-ventricular delay optimization on the intra left ventricular delay in cardiac resynchronization therapy

**DOI:** 10.1186/1476-7120-4-5

**Published:** 2006-01-26

**Authors:** Christoph Melzer, Fabian Knebel, Bruno Ismer, Hansjürgen Bondke, Christoph A Nienaber, Gert Baumann, Adrian C Borges

**Affiliations:** 1 Universitätsmedizin Berlin, Medical Clinic for Cardiology, Angiology, Pulmology, Charité Campus Mitte, Germany; 2 University of Rostock, Clinic for Internal Medicine, Rostock, Germany

## Abstract

**Background:**

Cardiac Resynchronization Therapy (CRT) leads to a reduction of left-ventricular dyssynchrony and an acute and sustained hemodynamic improvement in patients with chronic heart failure. Furthermore, an optimized AV-delay leads to an improved myocardial performance in pacemaker patients. The focus of this study is to investigate the acute effect of an optimized AV-delay on parameters of dyssynchrony in CRT patients.

**Method:**

11 chronic heart failure patients with CRT who were on stable medication were included in this study. The optimal AV-delay was defined according to the method of Ismer (mitral inflow and trans-oesophageal lead). Dyssynchrony was assessed echocardiographically at three different settings: AVD_OPT_; AVD_OPT_-50 ms and AVD_OPT_+50 ms. Echocardiographic assessment included 2D- and M-mode echo for the assessment of volumes and hemodynamic parameters (CI, SV) and LVEF and tissue Doppler echo (strain, strain rate, Tissue Synchronisation Imaging (TSI) and myocardial velocities in the basal segments)

**Results:**

The AVD_OPT _in the VDD mode (atrially triggered) was 105.5 ± 38.1 ms and the AVD_OPT _in the DDD mode (atrially paced) was 186.9 ± 52.9 ms. Intra-individually, the highest LVEF was measured at AVD_OPT_. The LVEF at AVD_OPT _was significantly higher than in the AVD_OPT-50_setting (p = 0.03). However, none of the parameters of dyssynchrony changed significantly in the three settings.

**Conclusion:**

An optimized AV delay in CRT patients acutely leads to an improved systolic left ventricular ejection fraction without improving dyssynchrony.

## Background

Asynchronous myocardial contraction in heart failure is associated with poor prognosis. Recent studies have shown an acute and sustained hemodynamic improvement after biventricular pacing (BVP), reversal of LV-remodelling, an increased quality of life, a reduction of symptoms of heart failure, and an improvement of exercise tolerance [1-7].

The optimization of the AV delay in DDD pacemaker patients is generally recommended and is performed in clinical practice. A variety of invasive and non-invasive methods were assessed in the past [8-15]. Recent studies have shown that also in CRT patients, invasively (dP/dt) [16-19] and non-invasively measured hemodynamic parameters (stroke volume) [20,21] are modified according to the programmed AV delay. A hemodynamically optimal AV delay can be defined.

Ismer's method of AV delay optimization [22] is validated for biventricular as well as right ventricular DDD pacing.

Tissue Doppler Imaging (TDI) is an evaluated tool in clinical practice to identify myocardial dyssynchrony. TDI (including strain and strain rate) imaging measures regional wall motion velocities and can accurately quantify regional left ventricular function [24].

Strain measures compression and distension of myocardial segments ("deformation imaging") and strain rate imaging expresses strain changes per time interval [25]. TSI (Tissue Synchronization Imaging) utilizes color-coded time-to-peak tissue Doppler velocities and visualizes segments of dyssynchrony in real-time by superimposing these temporal motion data on 2D echo images. [26,27].

These new techniques could potentially improve patient selection and guidance of implantation and programming of the devices for BVP. There is a variety of methods to determine dyssynchrony as summarized elsewhere [28].

There are no published data on the correlation of parameters of dyssynchrony and programming of the optimal AV interval. Aims of our study were therefore to investigate the influence of an optimized AV delay determined by the method of Ismer et al. [22] on dyssynchrony.

## Methods

### Patients

11 chronic heart failure patients of our clinic were included in this study. All patients had a biventricular ICD (pre-implantation NYHA III-IV, EF < 35%, QRS width > 120 ms). Clinical characteristics are demonstrated in Table 1. Patient exclusion criteria were as follows: atrial fibrillation, pacemaker malfunction and oesophageal diseases, NYHA IV, prosthetic mitral valve replacement.

**Table 1:** Patient characteristics

Age_(mean ± SD)_	63.2 ± 11.7
Gender (m/f) _(n/%)_	7(63.6)/4(36.4%)
Coronary artery disease _(n/%)_	4 (36.4%)
Dilated Cardiomyopathy _(n/%)_	7 (63.6%)
Left-ventricular ejection fraction _(mean ± SD)_	27.3% ± 11.9
Interval in months between stress testing and ICD implantation (months) _(mean ± SD)_	11.9 ± 12.9
location of the CS – electrode	
lateral	6 (54%)
posterolateral	4 (36.4%)
anterolateral	1 (9%)
diabetes mellitus _(n/%)_	6 (54%)
medication	
ACE inhibitors _(n/%)_	9 (82%)
ARB _(n/%)_	2 (18%)
Beta-blockers _(n/%)_	10 (91%)
Digitalis _(n/%)_	8 (73%)
Diuretics _(n/%)_	11 (100%)
spironolactone _(n/%)_	8 (73%)

ARB = Angiotensin-receptor blockers;

CS = coronary sinus

### AV delay: components and optimization

For the AV delay optimization we used the method proposed by Ismer et al [22].

**Figure 1:**
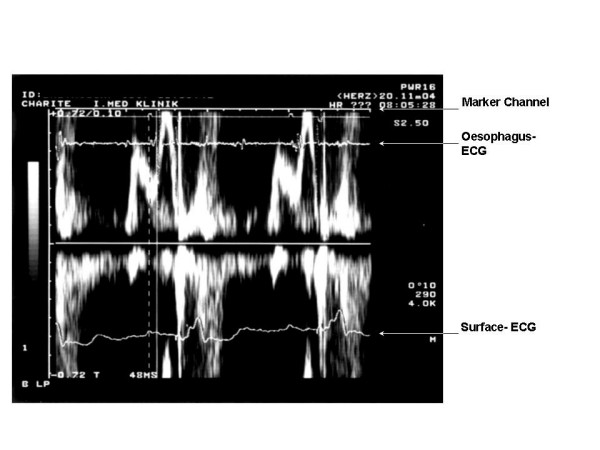
Measurement of the IACT in the VDD - Mode = MA-LA. MA = right atrial sensing marker (see marker channel). LA = left atrial deflection (see oesophageal ECG). In this particular patient the IACT is 48 ms.

**Figure 2:**
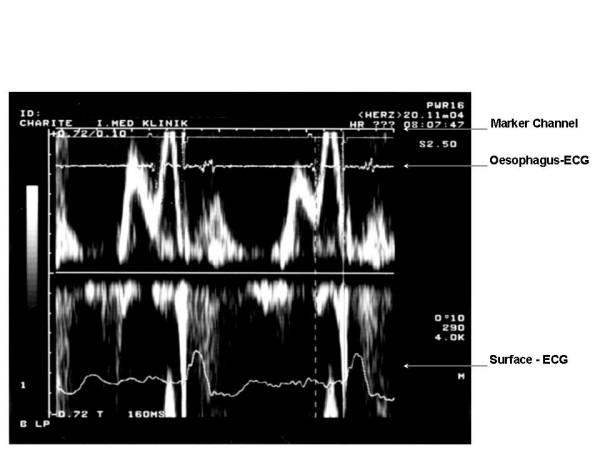
Assessment of the left-atrial electromechanical action = LA-EAC_long_. LA = left atrial deflection (see oesophagus- ECG). EAC_long _= the end of the A-wave in an unphysiologically long AV-intervall. In this particular patient the LA-EAC_lang _is 160 ms

This approach needs the placement of a bi-polar oesophageal electrode to provide a filtered left-atrial electrogram (LAE). We applied a 5F oesophagus electrode (Osypka TO2/5F, order no. TA12991101, Rheinfelden, Germany). Filtered oesophageal electrogram and telemetric real-time pacemaker markers provided by the programmer's analogue output were superimposed on the display of transmitral flow velocity on the Doppler-echo system (Figure 1). The simultaneous recording of transmitral flow, the left atrial oesophageal electrogram and the real-time sense-event markers, allow determining the components of the optimal AV delay (Table 2, Figure 1 and 2).

**Table 2:** Measurement of the components of the optimal AV delay according to Ismer et al. [22]

pacemaker-related interatrial conduction interval (IACT)	VDD pacing: MA-LA measured between right-atrial sense-event marker (MA) and the beginning of left-atrial deflection (LA) in oesophageal electrogram
	DDD pacing: SA-LA measured between right-atrial pacing stimulus (S_A_) and the beginning of left-atrial deflection (LA) in oesophageal electrogram
left-atrial electromechanical action (LA-EAC_long_)	Measured during unphysiologically long programmed AV delay between the beginning of left-atrial deflection (LA) in oesophageal electrogram and the end of the left-atrial contribution (EAC) in transmitral flow.
left-ventricular electromechanical latency period (Sv-EAC_short_)	Measured during unphysiologically short programmed AV delay between ventricular pacing stimulus (Sv) and the end of the left-atrial contribution (EAC) in transmitral flow.

Based on these measurements, optimal AV delays were calculated for VDD (atrial-triggered) and DDD (atrial-paced) mode using the equations:

AVD_OPT _VDD = MA-LA + LA-EAC_long _- Sv-EAC_short_

and

AVD_OPT _DDD = SA-LA + LA-EAC_long _- Sv-EAC_short_

### Echocardiograhy

Echocardiography to assess dyssynchrony was performed subsequently under three pacemaker settings: optimal AV delay (AVD_OPT_), optimal AV delay minus 50 ms (AVD_OPT_-50), optimal AV delay plus 50 ms (AVD_OPT_+50).

Echocardiography was performed on the Vivid 5 and Vivid 7 Dimension (GE Vingmed Ultrasound, Horton, Norway) machines. The TDI and strain analysis were performed in an off-line work station. The LVEF was assessed by area-length method in the apical four chamber view. The CI and the SV were calculated from the systolic velocities measured by PW-Doppler in the aortic outflow tract. Strain rate, tissue Doppler velocities were measured in the basal segments of the apical four-, three- and two-chamber views.

### Statistics

Values are expressed as mean ± standard deviation (SD). Groups were compared by parametric or non-parametric tests (t-tests and Wilcoxon-Mann-Whitney tests, respectively). Statistical significance was assumed at a value of P < 0.05. Statistical analysis was performed with the SPSS 12 software package (SPSS; Chicago, Ill, USA).

## Results

### Optimal AV delay

In all patients, we could define an optimal AV delay in the VDD and the DDD modes respectively. The AVD_OPT _in VDD mode was 105.5 ± 38.1 ms and the AVD_OPT _in the DDD pacing mode was 186.9 ± 52.9 ms. The results are summarized in Table 3. As expected, the mean optimal AV delay was lower in the VDD than in the DDD mode.

**Table 3:** AVD_OPT _VDD = optimal AV delay for atrially triggered (VDD) and atrially paced (DDD) modes

patient	AVD_OPT _VDD	AVD_OPT _DDD
1	60	172
2	78	154
3	96	204
4	92	132
5	92	144
6	122	174
7	136	216
8	64	128
9	84	180
10	168	252
11	168	300
	105,5 ± 38,1 ms	186,9 ± 52,9 ms

ARB = Angiotensin-receptor blockers; CS = coronary sinus

Echocardiography was performed subsequently under three pacemaker settings: AVD_OPT_, AVD_OPT_-50, AVD_OPT_+50. All patients had continuous biventricular stimulation even under AVD_OPT_+50.

### 2D and TDI echocardiography

The LVEF with AVD_OPT _was 28% (± 12%), with an AVD_OPT_-50 20% (± 7%, p= 0.03 compared to AVD_OPT_), with an AVD_OPT_+50 23% (± 7%, p = 0.11 compared to AVD_OPT_). The heart rate did not change significantly in the different settings (AVD_OPT_: 65,4/min, AVD_OPT_-50: 65,6/min, AVD_OPT_+50: 65,8 ms). The hemodynamics (SVI, CI, LVEF) and the TDI derived data are listed in Table 4. There was no significant difference of the amount of segments with dyssynchrony in TSI in the three settings. The maximal delay in the basal segments in the apical two-, three- and four-chamber views measured by TSI and strain did not differ in the AVD_OPT_, AVD_OPT_+50 and AVD_OPT_-50 setting.

**Table 4:** Hemodynamic and Tissue Doppler Echocardiography parameters in the AVD_OPT_, AVD_OPT_-50 and AVD_OPT_+50 modes.

Hemodynamics				
	AVD_OPT_	AVD_OPT_-50	AVD_OPT_+50	p
SV [ml]	89,2 (± 27.7)	89,7 (± 36.9)	95,3 (± 36.9)	n.s.
LVEF	0,28 (± 0.12)	0,20 (± 0.07)*	0,23 (± 0.07)**	*0.03 / **0.11
CI	3,0 (± 0.9)	3,2 (± 0.8)	3,4 (± 0.9)	n.s.
HR	65,4 (± 8.8)	65,6 (± 9.4)	65,8 (± 9.4)	n.s.
Tissue Doppler				
	AVD_OPT_	AVD_OPT_-50	AVD_OPT_+50	p
TDI max. delay in basal segments [ms]	122,9 (± 95,6)	125,0 (± 109,2)	131,7 (± 85,2)	n.s.
TSI segments with asynchrony	2,08 (± 1,24)	2,27 (± 1.19)	2,41 (± 1.43)	n.s.
Strain max. delay in basal segments [ms]	148,3 (± 74.9)	167,5 (± 90.2)	151,7 (± 54.1)	n.s.
Strain [%]				
4AC lateral	13,7 (± 6,5)	14.5 (± 9.8)	13,4 (± 9.8)	n.s.
4 AC septal	15,6 (± 7,3)	13.9 (± 9.3)	15.0 (± 9.8)	n.s.
A2C anterior	16,8 (± 9,7)	17.2 (± 10.5)	16.1 (± 9.1)	n.s.
A2C inferior	20,0 (± 11,5)	15.0 (± 11.4)	16.1 (± 9.8)	n.s.
A3C anterior	19,7 (± 9,5)	12.4 (± 9.7)	18.1 (± 9.8)	n.s.
A3C interior	19,1 (± 9,8)	14.1 (± 12.7)	19.2 (± 10.1)	n.s.

## Discussion

### Optimal AV delay

To date, Ismer's method for the optimal AV delay was applied to patients with DDD pacemakers and normal left ventricular function [22,23]. This is the first study to assess the optimal AV delay by Ismer's method in patients with reduced left ventricular function. In our CRT patients, an optimal AV delay according to Ismer's method could be defined. This is the only method that allows separate measurement of the three AV-delay components: i.e., the pacemaker-related interatrial conduction time, the left-atrial electromechanical action, and the left-ventricular latency period. The benefits of this method, however, are offset by the necessity for placement of an oesophageal electrode. This requirement explains why only a few medical centres have applied this method in clinical practice and in most cases for purposes of scientific investigation only.

Our results concerning the AVD_OPT _in the VDD mode (105.5 ± 38.1 ms) are in agreement with the results of other studies on AVD_OPT _in CRT patients: Butter [16] determined an AVD_OPT _of 100 ms in 30 patients, Auricchio [17] an AVD_OPT _of 112 ± 33 ms in 41 patients and Kass [18] an AVD_OPT _of 125 ± 49 ms. A study that was recently published by Porciani [29] found an AVD_OPT _during simultaneous biventricular pacing of 97 + 27 ms.

In the literature, there are no published data on AVD_OPT _in DDD mode. Therefore, our AVD_OPT _in DDD mode of 186.9 ± 52.9 ms cannot be compared to other studies.

### Hemodynamics

Intra-individually, the patients had the best LVEF under optimal AV-delay compared to the +50 and -50 ms settings. The LVEF is significantly higher in the AVD_OPT _setting than in the AVD_OPT _-50 setting. Obviously the formation of "cannon waves" seen with a shorter AV interval (AVD_OPT _-50) had a more negative hemodynamic effect than the diastolic mitral regurgitation seen with longer AV delays (AVD_OPT _+50). The hemodynamically unfavourable effects of "cannon waves" are described since the beginning of pacemaker therapy and are also termed "pacemaker syndrome". It is generally accepted that an adequate pacemaker programming can avoid this [30]. Toda et al. [31] could show in his studies that the mean LVEF in AVD_OPT _is higher than in prolonged AV delays. However, he found no significant difference.

### Dyssynchrony

Changes of dyssynchrony can be seen immediately, as seen in studies that have examined on/off comparisons in CRT patients [32]. However, an optimized AV interval does not change the markers of dyssynchrony. The reason for the improved hemodynamic situation under AVD_OPT _seems to be the better left ventricular filling and not the altered dyssynchrony.

## Limitations

This study included only a small number of patients. There was no follow-up examination of the patients.

## Conclusion

This study confirmed that an optimized AV delay improves the left ventricular ejection fraction. Acutely, the optimized AV delay does not influence left ventricular dyssynchrony. Whether a long-term AVD_OPT _leads to changes in left ventricular dyssynchrony via an improved LVEF and reverse remodelling can only be speculated. This has to be addressed in future studies with a long-term observation interval.

## Abbreviations

AVD_OPT _optimal AV delay

AVD_OPT_-50 optimal AV delay -50 ms

AVD_OPT_+50 optimal AV delay + 50 ms

CRT Cardiac Resynchronization Therapy

DCM Dilated Cardiomyopathy

EMD Electromechanical Delay

IVMD Inter-ventricular mechanical delay

LBBB Left Bundle Branch Block

SRI strain rate imaging

TDI Tissue Doppler Imaging

TSI Tissue Synchronization Imaging

VDD atrially triggered mode

DDD atrailly paced mode

EAC the end of the A-wave

LVEF Left ventricular ejection fraction

## Competing interests

The author(s) declare that they have no competing interests.

## Authors' contributions

CM and FK have equally contributed to this publication. CM, BI, FK and ACB have designed and performed the study and have written the manuscript. HJB, CAN and GB have participated in the study design and coordination and have helped to draft the manuscript. All authors read and approved the final manuscript.
